# Community Health Representative Workforce: Integration across systems and teams to address the social determinants of indigenous health and wellbeing

**DOI:** 10.3389/fpubh.2023.1047152

**Published:** 2023-03-15

**Authors:** Samantha Sabo, Louisa O'Meara, Janet Yellowhair, Joyce Hamilton, J. T. Neva Nashio, Brook Bender, Fernando Flores, Marianne Bennett, Rema Metts, Isabella Denton, Kim Russell

**Affiliations:** ^1^Center for Health Equity Research, Northern Arizona University, Flagstaff, AZ, United States; ^2^Hopi Tribe, Hotevilla, AZ, United States; ^3^White Mountain Apache Tribe, Whiteriver, AZ, United States; ^4^Hualapai Tribe, Peach Springs, AZ, United States; ^5^Colorado River Indian Tribe, Parker, AZ, United States; ^6^Salt River Pima Maricopa Indian Community, Scottsdale, AZ, United States; ^7^Gila River Health Care, Sacaton, AZ, United States; ^8^Arizona Advisory Council on Indian Health Care, Phoenix, AZ, United States

**Keywords:** Community Health Representatives, health systems, patient centered approaches, COVID-19, primary care, indigenous health and wellbeing, Community Health Workers

## Abstract

Tribally employed, Community Health Representatives (CHRs) serving Indigenous and American Indian and Alaskan Native (AIAN) peoples are culturally and linguistically embedded community leaders, with the unique ability to serve as the link and intermediary between community members and systems. Unique to the CHR workforce scope of practice is the expectation for high level integration within the medical and social service care team. This explicit role outlined in the scope of work sets an expectation for both CHR and care teams to deliver integrated patient, family, and systems level care coordination and case management. This paper aims to build from our previous manuscript published in Volume 1 of the special issue *Community Health Workers Practice from Recruitment to Integration*. In that Volume, we explored through a Community Case Study CHR Managers' perspectives on the challenges and opportunities for full CHR integration into health systems and teams serving AIAN. In this paper, we offer new information about the current CHR and CHR Managers' involvements and perceived level of integration within health care teams and the broader public health systems addressing the social and structural determinants of health. We approach this topic considering the COVID-19 pandemic and how CHRs and CHR Programs were included and not included in tribal pandemic response efforts.

## 1. Introduction

Tribally employed, Community Health Representatives (CHRs) serving Indigenous and American Indian and Alaskan Native (AIAN) peoples are culturally and linguistically embedded community leaders, with the unique ability to serve as the link and intermediary between community members and systems (1). Since 2015, community and academic partners from the Northern Arizona University Center for Health Equity Research (NAU-CHER), the Arizona Advisory Council on Indian Health Care (AACIHC), and 19 tribal Community Health Representative programs have come together to document CHR workforce roles and competencies (1, 2). Together, we have confirmed high levels of cultural, traditional, and linguistic experiences and knowledge held by the Arizona CHR workforce and its leadership, and the extant para-professional training they possess to meet the unique needs of AIAN patients and tribal and healthcare systems and teams. Unique to the CHR scope of practice, CHRs and their respective programs engage in case management and care coordination, including patient direct care, service coordination, patient navigation, and advocacy (3). These evidence-based characteristics are critical to high-functioning care teams, improved patient outcomes, and lower healthcare costs (4–6). Also unique to the CHR workforce scope of practice is the expectation for high level integration within the medical and social service care team (1, 7). This explicit role outlined in the scope of work sets an expectation for both CHR and care teams to deliver integrated patient, family, and systems level care coordination and case management. As a member of the care team, CHRs are expected to assist in the development of patient care plans, serving as both patient advocate and patient navigator to ensure continuity, completion, and acceptability of care. Yet, inherent challenges remain to optimize CHR integration, specifically in the areas of care team role delineation, communication, and coordination between care team providers (8, 9).

In Arizona, licensed healthcare providers, including those serving in Indian Health Service (IHS) and tribal health systems, consider CHRs to be valuable members of health teams (10). Moreover, Arizona Medicaid contracted health plans, 30% of whose members identify as Indigenous and AIAN, are highly motivated to integrate the broader Community Health Worker (CHW) workforce, inclusive of CHRs, within systems and teams (10). This is motivated in part by reforms in healthcare financing in the US, incentivizing a shift toward a value-based reimbursement structure that rewards evidence of favorable medical and social outcomes (11). Further evidence of the commitment and transition to patient centered coordinated care models to best serve Indigenous and AIAN populations, is observed through efforts by the Arizona Medicaid, known as the Arizona Health Care Cost Containment System (AHCCCS), to establish the American Indian Medical Home (AIMH) Program (12). Established in 2017, the AIMH Program, the first of its kind in the nation, was brought to fruition through a robust partnership between AHCCCS and tribal leadership in Arizona. The AIMH Program supports primary care case management, diabetes education, and care coordination for enrolled members. AIMH is intended to address health disparities between AIAN and other populations in Arizona by enhancing case management and care coordination. The AIMH is consistent with national moves of the IHS to adopt the Patient Centered Medical Home model, which IHS launched nationally in 2009 (13) and is currently operating in several tribally administered health systems across Arizona and beyond (14). In 2018, American Indian health policy entities in collaboration with Arizona Tribes advocated for the inclusion of CHRs as AIMH care team members. Despite a clearly defined CHR scope of practice within the health systems and primary care team, CHRs were not included as a designated reimbursable AIMH care team member by AHCCCS.

Most notable, is how tribally employed CHRs have been at the forefront of tribal communities' response to the COVID-19 pandemic. This experience illuminated both the greatest of potential and sorely missed opportunities for the entire CHW workforce, and specifically CHRs to be integrated into COVID-19 prevention and care systems to address serious health inequities laid bare by the “Merciless Monster” as the former Navajo Nation CHR Program Manager Mae-Gilene Begay MSW, once said (15). Throughout the pandemic and because of their trusted relationships and familiarity with the social and physical landscape of tribal lands and citizens, CHRs were invited to support public health surveillance, contact tracing, and case management of COVID-19 patients. CHRs provided critical health education and messaging around COVID-19 prevention and vaccination (7). Response efforts of Navajo Nation, White Mountain Apache Tribe and Hopi Tribe were nationally recognized for their effective, community-based infection prevention and mitigation strategies (15–18).

This paper is rooted in these experiences gained over the course of the pandemic, and knowledge and action related to growing evidence and policy opportunities in Arizona to implement best practices for integration of CHRs into systems and teams. Specifically, this paper aims to build from our previous manuscript published in Volume 1 of the special issue, *Community Health Workers Practice from Recruitment to Integration*. In that Volume, we explored through a Community Case Study CHR Managers' perspectives on the challenges and opportunities for full CHR integration into health systems and teams serving AIAN. In this paper, we offer new information about the current CHR and CHR Managers' involvements and perceived level of integration within health care teams and the broader public health systems addressing the social and structural determinants of health. We approach this topic considering the COVID-19 pandemic and how CHRs and CHR Programs were included and not included in tribal pandemic response efforts.

## 2. Context

Through a highly participatory process with major entities representing the interests of Indigenous and AIAN people throughout Arizona, and through funding from the CDC Community Health Workers for COVID Response and Resilient Communities (CCR), we launched the Community Health Representative Workforce Integration in Tribal Health Systems to Address COVID-19 (CHRs WITH uS!) project.

CHRs WITH uS! is a collaborative initiative, and one of just eight tribes, tribal organizations, or health service providers to tribes funded among the 69 organizations funded by the CDC CCR mechanism nationally. CHRs WITH uS! focuses on increasing the capacity of CHR Programs and their integration within the Indian Health Service and tribal health and care systems serving rural, Indigenous and AIAN citizens of Arizona. CHRs WITH uS! is led by the Arizona Advisory Council on Indian Health Care, in collaboration with a consortium of seven tribally operated CHR programs including: Cocopah Indian Tribe, Colorado River Indian Tribes, Gila River Health Care, Hopi Tribe, Hualapai Tribe, Salt River Pima-Maricopa Indian Community and White Mountain Apache Tribe with technical assistance and evaluation provided by Northern Arizona University, Center for Health Equity Research. In this community case study, we offer new insights afforded through the CCR grant held by CHR Programs regarding the roles they played in the COVID-19 pandemic response efforts; and current attitudes, beliefs and behaviors related to their workforce and programmatic integration within public health and health care systems and teams.

## 3. Key programmatic elements

### 3.1. CHRs WITH uS! workforce assessment

Through highly participatory methods, a CHRs WITH uS! Workforce Assessment was developed to establish a CHR workforce baseline and enable evidence informed strategic planning and policy over time. In alignment with community based participatory evaluation (CBPE) and best practices in Indigenous evaluation and CHW engagement practices, the assessment incorporated mixed methods including a survey and structured conversational interviews. The survey is designed as an annual, online cross-sectional survey of CHRs and CHR managers of Arizona. Survey domains include: (1) Demographics (race and ethnicity, age, gender, employment history, education, licensure and certification, income); (2) Roles, competencies and activities; (3) Referrals and Care Coordination (tribal health programs, health care systems); (4) Professional Development and Training; (5) Integration into primary care teams (roles, team members, perceived integration, communication); (6) Levels of collaboration with tribal Health Programs and; (7) COVID-19 Response (emergency preparedness, testing, tracing, vaccine roll out). The workforce survey was developed, piloted and revised in collaboration with the CHRs WITH uS! partnership and the Arizona State University CDC CCR 2110 National Evaluation team. Survey items are adapted from several sources including previous CHR workforce surveys conducted in Arizona (7), the 2021 AzCHOW CHW Workforce Integration Readiness Assessment (19), the 2020 Louisiana CHW Workforce Study (20), CHW Core Consensus Project (21) and the CHW Common Indicators Project (22). Here we present preliminary descriptive analysis using SPSS software for quantitative analysis.

The survey is coupled with a semi-structured qualitative interview conducted in a conversational style—in person or *via* Zoom—with CHR managers. The interview guide explores project implementation, program function, health and human service system integration, engagement with process and outcomes evaluation, and CHR Program involvement with COVID-19 response efforts. Detailed notes were taken during interviews, and in the case of Zoom meetings, interviews were recorded and transcribed in summary form. Notes were then revised for clarity and flow and sent back to the interviewee for review and approval. All interview transcripts were entered into Atlas.ti Qualitative Analysis Software and coded according to question domain through a rapid analysis method.

Again, in line with tenants of CBPE, best practices in Indigenous evaluation and CHW engagement practices, results were shared back with CHRs and managers through mini reports, presentations, and popular education techniques. Through these processes, assessment results were interpreted and clarified, while recommendations and strategic planning were explored and operationalized.

### 3.2. Participant demographics and professional training

A total of 48 CHRs and 13 CHR managers/supervisors completed the survey. Respondents represented 10 different CHR Programs or urban Indian health centers operating in Arizona (Table 1). Nearly 80% of CHRs and CHR managers identify as American Indian or Alaska Native women. CHRs and managers were similar in average age of 46 and 45 years old, respectively, both representing a large range in age. In terms of time in current position, CHRs averaged 7.5 years, with a range of being newly hired as a CHR with <1 year to CHRs with more than 40 years of experience. This is compared to more than half of CHR managers who reported being in their position for 5 years or less. Nearly all surveyed CHRs work in full-time positions, with more than half reporting a salary of $35,000 or less per year. CHR managers reported higher salaries, more than half earning over $50,000 annually. Nearly three-quarters of surveyed CHRs (73%) and CHR Managers (69%) reported having attended some college or having achieved a 2-year associate degree. Professional development and preparation are a cornerstone of CHR Programs, written into their job descriptions, therefore the workforce was asked about which licensures or certifications they hold. Choosing from a dropdown list of options, as well as writing in any others that were not listed, most CHRs and managers are First Aid/Basic Life Support (77% among both groups) and CPR (71 and 62%, respectively) certified. Nearly half of all CHR respondents are Certified Nursing Assistants (CNA). Training not specific to, but important to mention, held by this workforce included Respiratory Therapy Tech, Dialysis Patient Care Tech, Phlebotomist, Dietary Manager, and Certified Lactation Counselor. Some CHR managers also had specialty training including RN and AADE Diabetes Educator certification. Such professional certification and cross training add value to the CHR programs and make them highly desirable in remote and rural regions in which they work.

**Table 1:** CHR workforce survey participant demographics.

	**CHR (*N* = 48)**	**CHR managers (*N* = 13)**	**Total (*N* = 61)**
Age, mean years, (range)	45.7 (20–73)	45.3 (33–76)	45.6 (20–73)
**Gender**			
Female	81.3% (39)	84.6% (11)	80% (50)
Male	16.7% (8)	15.4% (2)	16.4% (10)
Non-binary	2.1% (1)	0	1.6% (1)
Time in position, mean years, (range)	7.5 years (< 1 to >42)	5.3 (< 1 to >13)	7.025 (< 1 to >42)
**Race and ethnicity**			
American Indian or Alaska Native	95.9% (47)	84.6% (11)	87.9% (58)
White	2% (1)	38.5% (5)	9.1% (6)
Black/African American	–	7.7% (1)	1.5% (1)
Hispanic or Latino	6.3% (3)	23.1% (3)	9.8% (6)
Tribal member	93.8% (45)	76.9% (10)	90.2% (55)
**Annual salary**			
$10,000–25,000	19% (9)	0 (0)	14.8% (9)
$25,000–35,000	40% (19)	8% (1)	32.8% (20)
$35,000–50,000	25% (12)	23% (3)	24.6% (15)
$50,000–75,000	0 (0)	54% (7)	11.5% (7)
$75,000+	0 (0)	8% (1)	1.6% (1)
Prefer not to answer	17% (8)	8% (1)	14.8% (9)
Full time employment status	98% (47)	100% (13)	98.4% (60)
**Education**			
Less than high school degree	6.3% (3)	0 (0)	4.9% (3)
High school graduate or GED	20.8% (10)	0 (0)	16.4% (10)
Some college, but no degree	37.5% (18)	53.8% (7)	41% (25)
Associates degree (2-year)	35.4% (17)	15.4% (2)	31.1% (19)
Bachelors degree (4-year)	0 (0)	30.8% (4)	6.6% (4)
**Licensure/certification**			N = 61
First aid/basic life support	77.1% (37)	77% (10)	77.0% (47)
CPR Certification	70.8% (34)	62% (8)	68.9% (42)
Certified Nursing Assistant	47.9% (23)	23% (3)	42.6% (26)
Certified Medical Assistant	17% (8)	15% (2)	13.1% (8)
Family Spirit Certification	14.6% (7)	23% (3)	16.4% (10)
CHW voluntary certification	8.3% (4)	0 (0)	6.6% (4)
Diabetes Community Care Coordinator	4.3% (2)	0 (0)	3.3% (2)
Registered nurse	2% (1)	8% (1)	1.3% (2)
^**^ AADE Diabetes Educator (manager)	4.2% (2)	7.7% (1)	
^**^Licensed Practical Nurse (LPN)	0 (0)	14.4% (2)	
^**^Registered Dietician (RD)	0 (0)	7.7% (1)	

** Write-in response.

### 3.3. CHR roles and activities

Although not the focus of this paper, we want to highlight that CHR roles and activities were also assessed. CHR roles and scope are set by the Indian Health Services, *Indian Health Manual*, which defines the standards of practice for the entire workforce (23). Additionally, findings from previous assessments with the Arizona CHR workforce (7, 24) have confirmed that CHRs' scope of practice is aligned with the Community Health Worker Core Roles as identified by the CHW Core Consensus Project (21). Presented with a list of 19 roles and or activities based on both sources, the current workforce survey confirmed again that Arizona CHRs and managers engage or support the full scope of practice. In this assessment, more than 80% of CHRs indicated that their work includes case find/screen, health education, individual and community outreach, medical appointments (scheduling, maintaining, etc.), and promoting healthy lifestyles (e.g., nutrition, exercise, etc.). According to responses from CHR Managers, a large part of their work is community-focused, including advocating for patients or community (85%), individual and community outreach (92%), and promoting healthy lifestyles (100%).

### 3.4. CHR collaboration with public health systems

**Figure 1:**
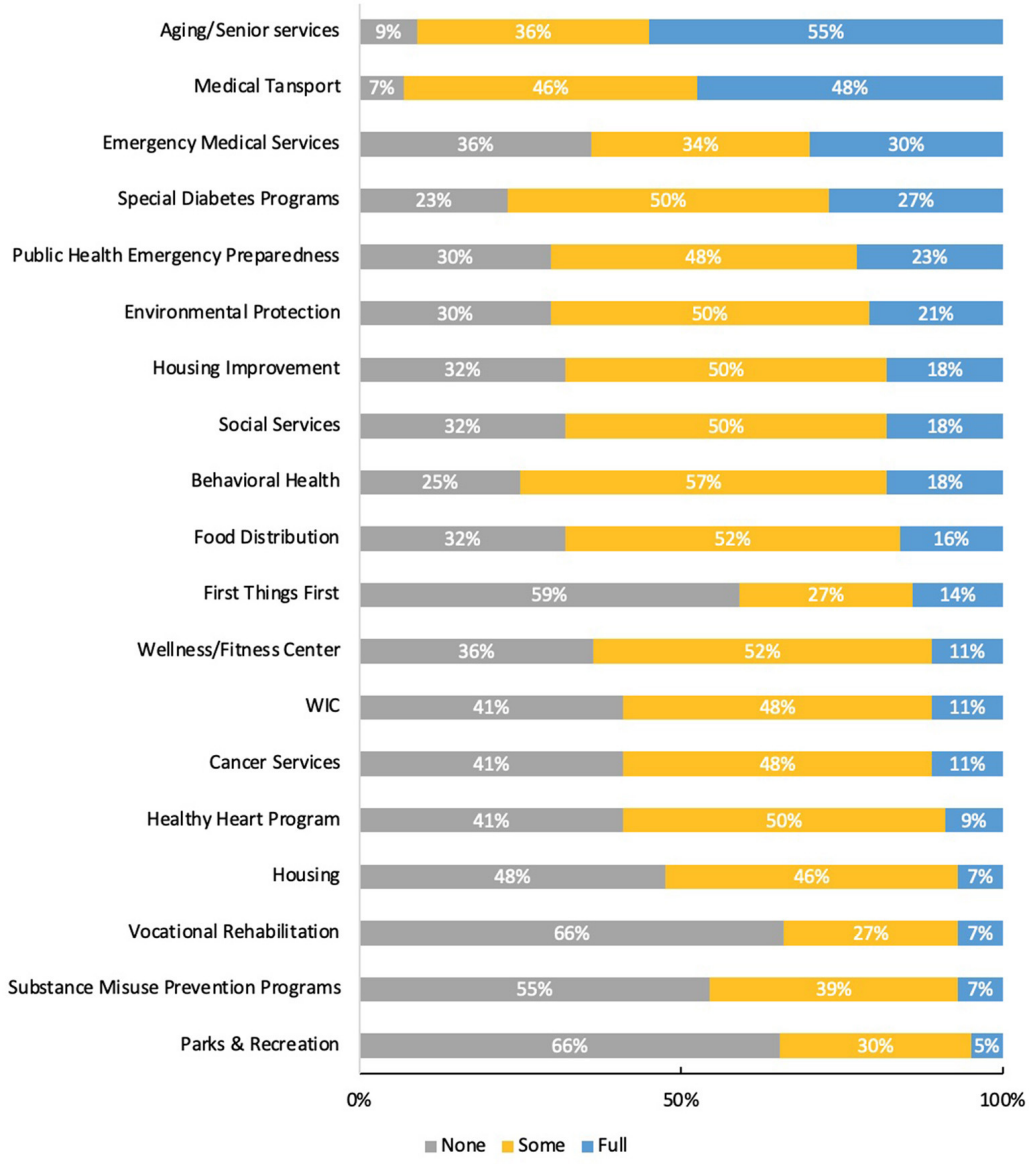
Collaboration among CHRs and public health services by type and level (*N* = 44 CHRs).

Fundamental to enacting the core CHR roles and competencies of cultural mediation, social support, advocacy and health education is the level of connection a CHR has to services and programs which address the social determinants of health (SDoH). We assessed the level (*none, some* or *full*) at which CHRs and managers collaborated with public health services available in their area and or operated by their Tribe or tribal organization. Levels are defined as no interaction (none), some interaction (send/receive referrals, occasional communication), and full collaboration (frequent communication, referrals, joint projects). Here we present CHR responses only (Figure 1). CHRs' experiences varied, with approximately half of all CHRs reporting *some* collaborative relationship with the following programs: medical transportation, the IHS-coordinated Special Diabetes Program for Indians (SDPI), housing, environmental protection, social services, behavioral health, and food distribution. Given the reverence and historical commitment of the national CHR Program to community elders, more than half of all programs reported having a *full* collaboration with aging and senior service programs operated in their communities. Transportation is a major structural determinant of health for many community members living on tribal homelands, which is reflected in the high level of reported *full* collaboration with medical transport services operated by the Tribe and IHS. Although the assessment results highlighted several opportunities for new partnerships, especially with vocational rehabilitation, parks and recreation and the First Things First initiative (a state-run program supporting parents and children aged 0–5 years), we recognize that not all of these types of services are available to all survey respondents, and therefore may have been reported as *none*.

### 3.5. CHR social determinants of health referrals

**Figure 2:**
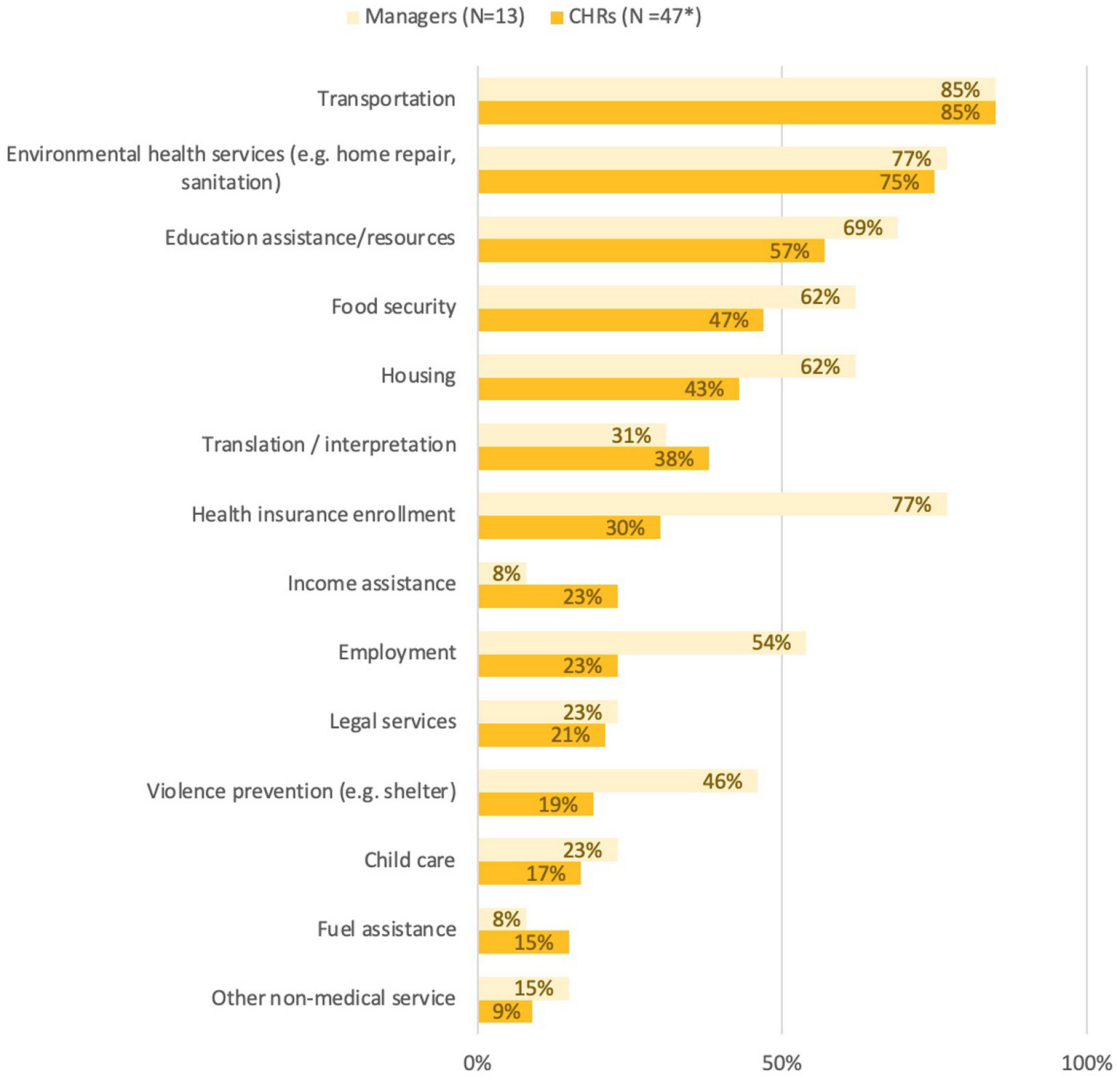
CHR referral categories.

Building from the level and type of collaborations that CHRs have with other community programs—we assessed how CHRs and managers address the social and structural determinants of health by connecting clients to services through referrals. The referral service categories included in the survey were generated through free listing with managers and CHRs of the known programs and services in their region, and further adapted from a recent Community Health Worker Workforce Study (20). Among both CHRs and CHR manager respondents, the two most common referral categories were transportation and environmental health services. Environmental health services are a broad category that was defined and interpreted as including home repair programs that address access to electricity, potable water, and sanitation services, as well as programs that address home safety and disability access such as installment of wheelchair accessible ramps. The largest differences (>20% margin) between CHRs' and managers' reported referral categories were found in health insurance enrollment, employment services, and violence prevention—with a significantly greater percentage of managers perceiving CHRs connecting clients to these three services. These data suggest programs are connecting community members to services critical to addressing the major SDoH of education, food, housing and language and interpretation services (Figure 2).

### 3.6. CHR program involvement in COVID-19 response

**Figure 3:**
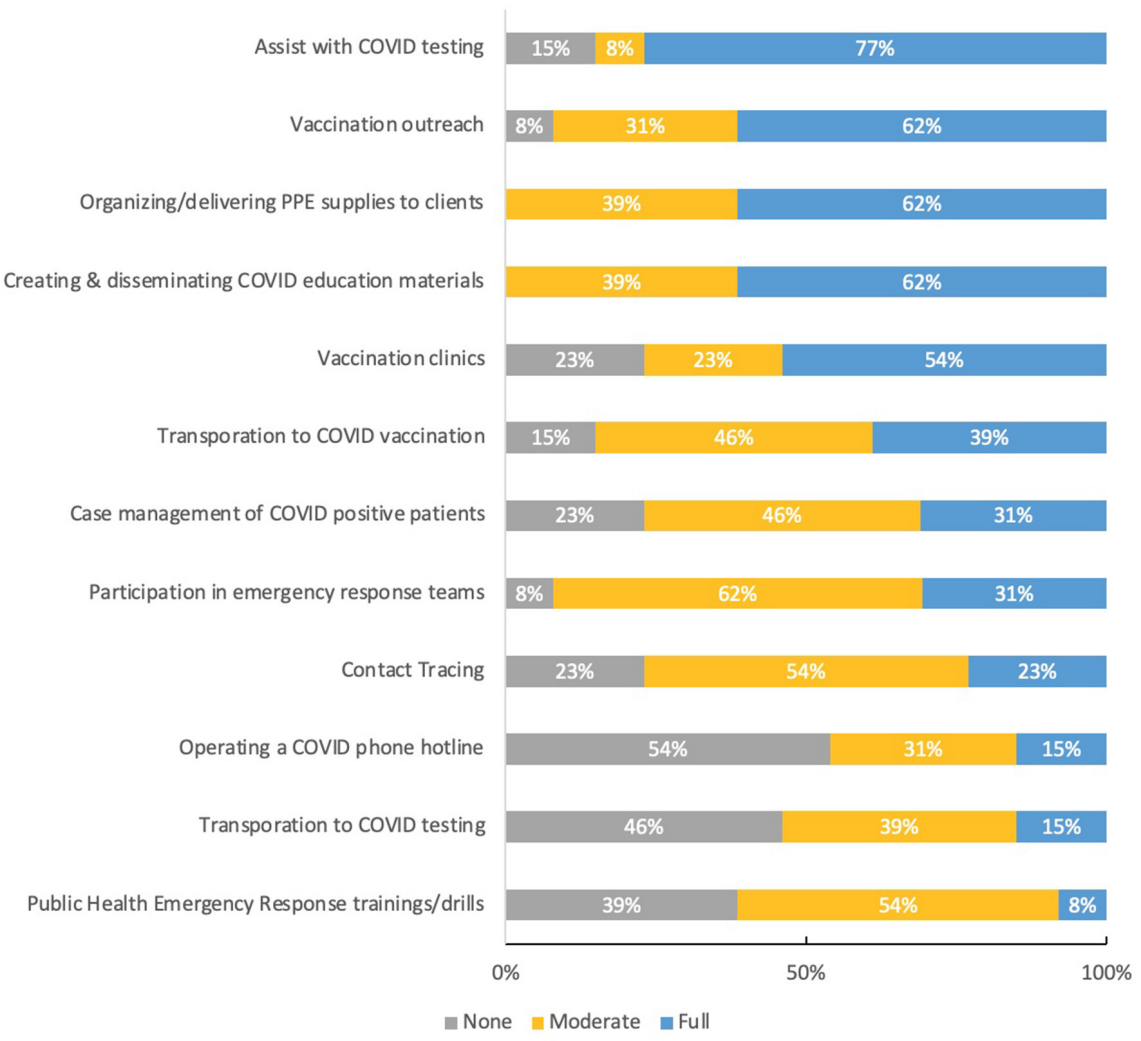
CHR manager descriptions of CHR program involvement in COVID-19 response efforts.

Next, we explore how CHR Programs were engaged in COVID-19 pandemic response efforts (Figure 3). We intentionally offer this information at this point in the community case study to set up the next section which explores how CHRs and programs are integrated into health care systems and teams currently.

COVID-19 demonstrated to the world how critical the CHW workforce, inclusive of CHRs, was in addressing COVID-19 inequities disproportionately experienced by Black, Indigenous and People of Color (BIPOC). As has been reported elsewhere, nationally, Indigenous and AIAN populations experienced higher age-adjusted COVID-19 mortality rates than any other racial or ethnic group (25), as well as a 1.6 × higher risk of infection and 3.5 × risk for hospitalization than non-Hispanic Whites (26). The COVID-19 incidence rate per 100,000 continues to be significantly higher among Indigenous AI/AN (70.01) compared to non-Hispanic Whites (24.97) (27). Indigenous and AIAN populations in Arizona have experienced a disproportionate impact from COVID-19. As May 2021 ~25% of *all* AIAN deaths in the U.S. having occurred in Arizona (1,596 out of 6,382) (28). AIAN deaths represented between 8 and 10% of all COVID-19 deaths in the state, in spite of AIAN populations being 5% of the total population (28, 29). Although this rate has fluctuated over the course of the pandemic; in May 2020 American Indian people comprised over 12% of cases and 16% of deaths in Arizona (30). These health disparities, in concert with historical and contemporary inequities rooted in lack of access to healthcare and running water, and crowded housing, have placed Indigenous and AIAN populations at greater risk for infection and severe outcomes of COVID-19 (26, 30, 31).

Given a list of COVID-19 response categories, identified through our previous workforce assessment, CHR Program Managers were asked to rate the degree to which their program has been involved in each effort—options were presented on a three-point scale of not involved, moderately involved (occasionally), and highly involved (daily/weekly).

According to our assessment, more than three-quarters of all CHR Managers described their program as highly involved (daily/weekly) in assisting with COVID-19 testing, while slightly less than two-thirds of programs were highly involved (daily/weekly) in vaccination outreach and creating and disseminating COVID-19 educational materials. Half of all programs were also highly involved (daily/weekly) in supporting vaccine clinics. Approximately 62% of CHR Programs were described as moderately involved (occasionally) involved in their Tribes' emergency response team and or participating in emergency response training and skills. In the following section, CHR Managers describe in detail the various activities they contributed to and in some cases led.

In qualitative interviews, manager experiences of their programs' integration into COVID response efforts fell into two major categories: CHR involvement ranges from minimal (e.g., involvement limited to distributing home testing kits) to essential (e.g., invited to lead incident Emergency Operation Center or lead testing and vaccine distribution).

#### 3.6.1. Minimal involvement in COVID response

The involvement of several CHR Programs in community COVID response was limited to distribution of home test kits and personal protective equipment (PPE). The first CHR Program manager explained that while their staff was trained to provide contact tracing, they were not invited to participate in contact tracing or incident command. CHR involvement was limited to providing home test kits and PPE (sourced through the National Supply Center) to residents during community testing events and through regular office services, and helping public health nurses identify unhoused residents for vaccination. Similarly, the main role that the second CHR Program played in COVID response was to distribute home test kits and deliver medicine to homebound clients. CHRs received contact tracing training and were initially invited to do some contact tracing and case management of positive clients but were eventually not included in this aspect of the response. Managers attributed some of their underutilization in response efforts to a lack of understanding from IHS of CHR roles and responsibilities. The third CHR Program's primary role in COVID response was to distribute COVID-related informational materials, home test kits and PPE kits to residents. IHS Public Health Nursing (PHN) and Tribal Emergency Management were responsible for testing and vaccination coordination, but due to staffing limitations and geographical challenges, they enlisted the support of a local federally qualified community health center (FQCHC) to provide both services on occasion. CHRs were brought in to support pop-up clinics, and to serve as a liaison between the FQCHC and IHS PHN.

#### 3.6.2. Invited to be essential members of the COVID response and or vaccination efforts

The fourth CHR Program director described their strong integration into the COVID response effort in their community. CHRs had already completed FEMA training before the pandemic began, so they were well positioned to be part of the response team. The CHR manager was made the head of the Operations section of the Emergency Operation Center and CHRs provided IHS staff with information about families and individuals in the community that was essential in determining health status, risk level, and living situation. CHRs worked with PHN to assist with vaccination and testing efforts, assisted with mass testing events held at various locations including the local casino, housing authority, daycare centers, and behavioral health services. CHRs also provided case management to positive clients, which includes a focus on identifying and monitoring high-risk household members. CHRs were trained for high-risk care and case management work with COVID-positive clients designated as high-risk by IHS.

The directors of the fifth and sixth CHR Programs described a similarly high level of involvement in their communities' COVID response. From the beginning of the pandemic, both CHR programs were included in a Joint Incident Command team that coordinated the COVID response between the Tribe and the tribally operated 638 hospitals. The fifth CHR Program was tasked with managing the Tribe's entire vaccination program, coordinating weekly mass vaccination clinics. The sixth CHR Program worked with PHN to lead COVID testing efforts, assisting with mass testing events that serviced as many at 400–600 people in a day during the height of the pandemic, and provided testing of residents of the skilled nursing facility. CHRs at that program were also involved in contact tracing and were included as “essential” members of the PHN-led home visiting vaccination teams, providing explanation about the vaccine to community members. The director of the sixth CHR Program described the influence the pandemic had on the overall focus of the CHR program, pushing it from primarily health education and disease prevention to medication management and support for high-risk clients. CHRs were critical in checking on high-risk, homebound COVID-positive residents, providing case management, delivering medication, assisting with medication management, and assessing their needs. Through their efforts, data was also collected to establish a long-term COVID clinic (for “COVID long-haulers”).

### 3.7. Integration in primary care systems and teams

Finally, we turn our attention to how the CHRs and managers perceive their current involvement in healthcare systems and teams. According to the workforce assessment, approximately, 60% of CHR managers and CHR respondents believe they are part of a primary care team, compared with 40% of respondents who reported no involvement or unsure (Table 2). Of those CHRs and managers who are part of a primary care team, more than 50% of all CHRs and managers described that PHNs, fellow CHRs, CHR managers, medical assistants, doctors, and pharmacists were part of the care team. Social workers, community members and patients, nutrition specialists and behavioral health counselors were included by less than half of all respondents as members of the care team. CHRs and managers were also asked which current modes of communication they utilize to communicate with members of the care team. Although respondents could choose all that apply, telephone messages and text were the primary form of communication. Approximately 67% of all respondents reported being involved in huddles or meetings with the care team. Major differences in perceived modes of communication occurred in relation to perceived access to an electronic health medical record, with 62% of CHRs believing they used an EHR to communicate with the team compared to only 36% of managers. Notably, approximately one-quarter and one-third of CHRs reported no formal way to communicate, in passing only or no way to communicate at all, respectively.

**Table 2:** CHR program integration within health systems and teams.

**Work as part of a primary care team**	***N*** **= 45**	***N*** **= 13**	***N*** **= 58**
Yes	60% (27)	61.5% (8)	60.3% (35)
No	20% (9)	23.1% (3)	20.7% (12)
Unsure	20% (9)	15.4% (2)	18.9% (11)
**Members of the primary care team**	***N*** = **36**	***N*** = **10**	***N*** = **46**
Registered Nurses/Public Health Nurses	91.7% (33)	90% (9)	91.3% (42)
CHR Mangers/Supervisors	83.3%(30)	60% (6)	78.3% (36)
Fellow CHRs	77.8% (28)	80% (8)	78.3% (36)
Medical Assistants	69.4% (25)	70% (7)	69.6% (32)
Doctors	66.7% (24)	80% (8)	69.6% (32)
Pharmacists	58.3% (21)	50% (5)	56.6% (26)
Social Workers	47.2% (17)	70% (7)	52.2% (24)
Community Members/Patients	44.4% (16)	20% (2)	39.1% (18)
Nutritionist/Dietitian	44.4% (16)	40% (4)	43.5% (20)
Behavioral Health Counselors	27.8% (10)	30% (3)	28.3% (13)
**Current modes of communication with primary care team**	***N*** = **42**	***N*** = **11**	***N*** = **53**
Telephone message and text	88.1% (37)	72.7% (8)	84.9% (45)
Grand rounds, huddles, meetings	66.7% (28)	63.6% (7)	66.0% (35)
Handwritten notes	64.3% (27)	63.6% (7)	64.2% (34)
Resource patient management systems	64.3% (27)	36.4% (4)	58.5% (31)
Electronic health record (EHR)	61.9% (26)	36.4% (4)	56.6% (30)
Medical chart	61.9% (26)	45.5% (5)	58.5% (31)
No formal way, in passing only	35.7% (15)	9.1% (1)	30.2% (16)
No way of communication	23.8% (10)	9.1% (1)	20.8% (11)
**Perceptions of integration**	***N*** = **45**	***N*** = **13**	***N*** = **58**
**I feel I am a valid member of the primary care team**			
Strongly agree	24.4% (11)	15.4% (2)	22.4% (13)
Agree	55.6% (25)	61.5% (8)	56.9% (33)
Disagree	15.6% (7)	15.4% (2)	15.5% (9)
Strongly disagree	4.4% (2)	7.7% (1)	5.2% (3)
**I feel I am well integrated into the primary care team**			
Strongly agree	13.3% (6)	15.4% (2)	13.8% (8)
Agree	57.8% (26)	46.2% (6)	55.2% (32)
Disagree	26.7% (12)	23.1% (3)	25.9% (15)
Strongly disagree	2.2% (1)	15.4% (2)	5.2% (3)
**I feel the healthcare providers I interact with have a good understanding of my roles and abilities**			
Strongly agree	26.7% (12)	15.4% (2)	24.1% (14)
Agree	57.8% (26)	61.5% (8)	58.6% (34)
Disagree	15.6% (7)	15.4% (2)	15.5% (9)
Strongly disagree	0 (0)	7.7% (1)	1.7% (1)

How CHRs and managers feel as members of the primary care team was also explored. This question was asked of all respondents, not only those who identified as members of a primary care team. A four-point Likert scale (*strongly agree, agree, disagree*, and *strongly disagree*) was used to assess three questions. Generally, one-quarter of respondents *strongly agreed* that they feel they are a valid member of the healthcare team and that healthcare providers they interact with have a good understanding of their roles and abilities. These trends tracked for those respondents who *agreed* (as opposed to *strongly agreed*) with these statements, with more than 50% of respondents stating they *agreed*. When asked if CHRs and managers feel they are well integrated into the primary care team, the level of *strongly agreed* responses dropped to 13 and 15% for CHRs and managers, respectively. Also notable, is the significant difference between CHRs and managers who *agreed* with this statement, 57 and 46%, respectively. Overall, although results trend positively, they also demonstrate opportunities to improve the level of integration among CHRs and managers within systems and teams.

## 4. Discussion

The CHRs WITH uS! workforce assessment is a set of powerful tools to generate workforce informed systems-level approaches to monitor progress toward a variety of workforce identified goals and aims related to public health and health care systems and care team integration, including COVID-19 related response systems and teams.

In pursuit of the realization of the full CHR scope of practice and enabling CHRs and all team members to practice at the top of their scope, McCarville et al. (32) identified several health systems factors associated with the quality of integration of CHWs into systems and teams. According to this model, at the health systems level, our workforce assessment identified several of these factors. We found evidence of moderate to high levels of the following factors: respondent reported working as part of a care team; mechanisms exist for CHRs and care teams to communicate; CHRs work in close physical proximity to care team members (share physical workspaces). We also identified moderate to low levels of the following factors that contribute to quality integration: CHRs having access to EMR or other medical record systems; and having a known champion or leader within the team that supports CHR integration. We found low to no evidence of the following factors: healthcare providers receive training or mentorship in working with CHRs; and protocols and procedures involve CHRs in health services delivery. What is currently unknown and yet to be explored are the final health system factors of: protocols that guide CHR participation in regular meetings with care team; and a flattened hierarchy enabling CHRs to engage in aspects of care.

### 4.1. Workforce policy recommendations

Over the course of the CHRs WITH uS! project, and through the efforts of the broader Arizona CHR Workforce Movement (coalition), CHR Programs have decided to engage in a Program-to-Program Mentorship (PPM) program. PPM will pair or match CHR programs that have self-identified to have demonstrated strengths, protocols, or policies in integration within systems, care coordination and closed loop referral systems development, or have experienced high level engagement within COVID-19 response efforts, with CHR programs without such experience but with the desire to engage. PPM intends to build from local knowledge, lessons learned and processes operating within CHR Programs and their related IHS and tribal systems of care. We believe such a model may increase the likelihood of adoption of systems and team integration by creating space for broader systems-level leadership and team members to engage directly through trusted channels. This direct engagement is opposed to seeking a model from outside or from a context without the level of trusted relationships or proximity required to implement new strategies over time.

CHRs WITH uS! partners and broader consortium members are currently focused on formalizing relationships with tribal programs and health systems that include: (1) establishment of formal referral process and procedures to improve communication between CHRs and IHS, (2) access to electronic health records for CHRs, (3) participation in discharge planning for clients returning to communities, and (4) formal case management policies and procedures. Partners have identified several mechanisms to integrate CHRs into systems and to fully utilize their scope of practice to benefit and address the social determinants of health and resilience with their clients.

### 4.2. Conceptual or methodological constraints

This community case study is not considered research by Northern Arizona University Institutional Review Board. It is not intended to be generalizable to the broader CHR workforce and is unique to the tribal CHR Programs and Urban Indian Health Centers employing CHRs within the boundaries of the state of Arizona. Despite the non-generalizability, assessment methods were conducted in highly participatory ways with workforce and management involved at each phase of the assessment including conceptualization, instrumentation, interpretation of results and dissemination of results.

## Data availability statement

The datasets presented in this article are not readily available due to Tribal Data Governance. Requests to access the datasets should be directed to samantha.sabo@nau.edu.

## Author contributions

SS and LO'M lead the writing of the community case study. KR provided detail review and supported the development of public health policy recommendation. BB, JH, JN, FF, MB, RM, and JY participated in the development of the workforce assessment protocol and including its focus and conversation guides. All authors contributed to the interpretation of workforce assessment results and provided detailed review of draft reports and reviewed the final versions of the community case study. All authors co-conceptualized the community case study based on the original community health representative workforce assessment results and reports. All authors contributed to the article and approved the submitted version.
